# Genome-wide QTL and eQTL mapping reveal genes associated with growth rate trait of the Pacific white shrimp (*Litopenaeus vannamei*)

**DOI:** 10.1186/s12864-024-10328-9

**Published:** 2024-04-26

**Authors:** Xiuli Chen, Min Peng, Chunling Yang, Qiangyong Li, Pengfei Feng, Weilin Zhu, Yongde Zhang, Digang Zeng, Yongzhen Zhao

**Affiliations:** Guangxi Key Laboratory of Aquatic Genetic Breeding and Healthy Aquaculture, Guangxi Academy of Fishery Sciences, Nanning, 530021 China

**Keywords:** Genetic map, QTL, eQTL, Growth rate trait, *Litopenaeus vannamei*

## Abstract

**Background:**

Growth rate is a crucial economic trait for farmed animals, but the genetic regulation of this trait is largely unknown in non-model organisms such as shrimp.

**Results:**

In this study, we performed genome-wide phenotypic quantitative trait loci (QTL) and expression quantitative trait loci (eQTL) mapping analyses to identify genes affecting the growth rate of Pacific white shrimp (*Litopenaeus vannamei*), which is the most commercially-farmed crustacean worldwide. We used RNA-sequencing of 268 individuals in a mapping population, and subsequently validated our findings through gene silencing and shrimp growth experiments. We constructed a high-density genetic linkage map comprising 5533 markers spanning 44 linkage groups, with a total distance of 6205.75 cM and an average marker interval of 1.12 cM. Our analyses identified 11 QTLs significantly correlated with growth rate, and 117,525 eQTLs. By integrating QTL and eQTL data, we identified a gene (metalloreductase *STEAP4*) highly associated with shrimp growth rate. RNA interference (RNAi) analysis and growth experiments confirmed that *STEAP4* was significantly correlated with growth rate in *L. vannamei*.

**Conclusions:**

Our results indicate that the comprehensive analysis of QTL and eQTL can effectively identify genes involved in complex animal traits. This is important for marker-assisted selection (MAS) of animals. Our work contributes to the development of shrimp breeding and available genetic resources.

**Supplementary Information:**

The online version contains supplementary material available at 10.1186/s12864-024-10328-9.

## Background

Many important economic traits in animals, including growth rate, are complex traits controlled by quantitative trait loci (QTL) [1]. QTL analysis is an effective method to identify genomic regions controlling specific traits, which can further be used to find candidate genes or molecular markers linked to the traits for marker-assisted selection [2]. However, in the past, QTL studies were based on a limited number of markers with limited mapping resolution, resulting in the detection of QTL regions that typically contained thousands of candidate genes [3]. To narrow down the boundaries of target QTL and precisely locate candidate genes, expensive, laborious, and time-consuming fine mapping and positional cloning strategies are often required [4]. The introduction of next-generation sequencing (NGS) technologies has made it easier to develop hundreds of thousands of single nucleotide polymorphism (SNP) markers. The combination of large-scale data generated by NGS and powerful computational tools has resulted in significant technological advancements in QTL mapping from low to high resolution [5]. After fine mapping, the number of candidate genes within the QTL can be reduced to hundreds [6]. However, identifying the causal genes for the traits remains a major challenge due to the expensive and time-consuming procedures involved in validating the candidate genes [7]. This limitation greatly restricts the study of quantitative traits. Furthermore, this independent analysis alone cannot fully explain the genetic regulatory mechanisms underlying complex traits. Recently, a method for identifying candidate genes, known as expression quantitative trait locus (eQTL) analysis or genetical genomics, has been developed [8]. eQTL analysis applies genetic linkage analysis of whole-genome expression patterns obtained through microarray or NGS technologies to study genetic variation in gene expression. In eQTL analysis, mRNA transcript abundance is treated as a quantitative trait and mapped as eQTL [9]. eQTL analysis can construct gene regulatory networks to further investigate the coordinated functions of genes controlled by common eQTLs, thereby elucidating the genetic basis of complex traits at the gene expression regulation level [10]. eQTL has been applied in studies across multiple species and has been proven to be an effective method for identifying candidate genes [11–13].

Growth rate is one of the most important economic traits in aquaculture animals and is closely related to the economic benefits of aquaculture. Although the growth rate trait in shrimp is polygenically controlled quantitative trait, the genetic regulatory mechanism underlying this trait is still largely unknown [14]. Recently, some studies have reported the construction of genetic linkage maps and identification of QTLs for the growth rate trait in shrimp [15–17]. However, these QTL intervals contain a large number of candidate genes, and the major regulatory genes for the growth rate trait remain unknown [18].

Shrimp are economically important aquaculture animals that provide a significant protein source for humans, while also playing essential ecological roles in marine and freshwater environments. The Pacific white shrimp (*L. vannamei*) is the most globally important species, accounting for over 50% of total shrimp production [19]. To gain deeper insights into the genetic regulatory mechanisms of growth in *L. vannamei* and search for candidate genes associated with growth rate trait, we constructed a genetic linkage map of *L. vannamei* using RNA sequencing. For the first time, we explored candidate genes related to growth rate in *L. vannamei* through integrated analysis of phenotypic quantitative trait loci (QTL) and eQTL. Candidate genes associated with growth were subsequently identified and validated through gene silencing and shrimp growth experiments. This study provides critical new genomic information for shrimp breeding and investigations of *L. vannamei*. The method outlined in this study for identifying candidate genes through comprehensive analysis of QTL and eQTL may also provide valuable preferences for other species.

## Methods

### Preparation of mapping family for eQTL analysis

The shrimp used in this experiment were obtained from Guangxi Shrimp Breeding Engineering Technology Research Center (Nanning, Guangxi, China). The mapping family was created through artificial insemination. To elaborate, a female shrimp and a male shrimp from a family achieved through eight generations of full-sib matings, originally derived from an SPF strain obtained from the Hawaii Institute of Oceanography) were mated. The resulting offspring comprised the mapping family. All of these offspring were cultured together in an indoor pool measuring 12 m×6 m and were fed a standard diet. After five months of cultivation, the family, consisting of two parents and their 268 offspring, was collected for genetic mapping. The hepatopancreas tissue was promptly removed from each shrimp and stored in liquid nitrogen for subsequent RNA extraction. The body weights of the shrimp were measured using an electronic scale.

### RNA extraction, library construction, and RNA-Seq

Total RNA was extracted from the hepatopancreas by grinding them in liquid nitrogen and using the TRIzol® LS Reagent (Ambion, US) following the manufacturer’s protocols. Genomic DNA was removed using DNase I (Ambion, US). The concentration of RNA in each sample was measured using a NanoDrop ND-2000 Spectrophotometer (Thermo Scientific, USA) and adjusted to equivalent concentrations across samples using ultrapure water. RNA quality was assessed using a Bioanalyser 2100 (Agilent, USA), and samples with an RNA Integrity Number (RIN) value > 7 were used to construct cDNA libraries for sequencing. The cDNA libraries for RNA-seq sequencing were prepared using the NEBNext Ultra RNA Library Prep Kit (Illumina, US) manufacturer’s the manufacturer’s instructions and sequenced on the Illumina HiSeq2500 platform.

### SNP identification and linkage map construction

The software STAR [20] was used to align clean reads from each sample to a full-length transcriptome dataset of *L. vannamei* that we previously constructed [21]. This alignment was performed to quantify the expression levels of each unigene. SNPs were identified using the GATK software program [22]. The identification criteria included no more than three consecutive single-base pair mismatches within a 35-bp region and a SNP Quality Score > 2.0 after sequence depth normalization. SNP labeling was conducted by removing all SNPs that had missing parental labels, parental depths less than 3x the labels, or non-polymorphic labels present between markers. In addition, SNPs with genotype coverage across all offspring less than 60% were removed. Polymorphic SNPs with significant segregation distortion (chi-square test *P* < 0.001) were also excluded. Finally, the selected SNP labels were incorporated into the HighMap software program [23]. This incorporation allowed for the calculation of MLOD values between each pair of labels. The minimum and maximum numbers of groups were set, the MLOD value ranges were preset, labels were arranged in ascending order based on their MLOD values, and labels with the highest MLOD values were grouped together into the same linkage group. The linear arrangement of the markers within each linkage group was analyzed, and the genetic distances between adjacent markers were estimated to obtain the final genetic map. A comparison between the map and a genome of *L. vannamei* (NCBI accession: JANIEY000000000) published by our team [24] was performed. A total of 4053 sequences from the map were compared to the 44 pseudochromosome of the genome using the Blastn program (http://blast.ncbi.nlm.nih.gov/Blast.cgi; v2.2.26 + x64-linux) with the e-value threshold of 1e^− 5^.

### Mapping of QTLs

QTL analysis was conducted using the body weight of *L. vannamei* as an indicator of growth rate. The Composite Interval Mapping method in the MapQTL 5.0 software was used for QTL mapping [25]. The significance thresholds of LOD scores were calculated based on 10,000 permutations with an experiment-wise significance level of < 0.05 QTLs with a maximum LOD score greater than or equal to the significance threshold were considered statistically significant. Confidence intervals corresponding to a LOD score decrease of one or two on either side of the likelihood peak were calculated for each significant QTL. The proportion of variance explained by a QTL peak (Expl) was calculated using the formula: $\text{E}\text{x}\text{p}\text{l}=1-{10}^{-2\text{L}\text{O}\text{D}/\text{N}}$ ,Where N is the number of samples [26].

### Mapping of eQTLs

The expression levels of genes were calculated by aligning them to the *L. vannamei* genome, and the mapped reads were used to calculate the expression profile for each shrimp sample. The expression of genes was represented by Reads Per Kilobase per Million mapped reads (RPKM) values. Only genes with complete expression (genes with RPKM = 0 were considered missing) of 80% or above were used for eQTL analysis. The eQTL mapping was conducted using the method similar to the QTL mapping analysis, but with gene expression data as the trait. The mapping was also analyzed using the MapQTL 5.0 software [25].

### Co-location of QTLs and eQTLs

When an eQTL for a gene was located within 5 cM upstream or downstream of the genetic distance of the gene, it was considered a cis-eQTL [27]. If it was outside of this range, it was considered a trans-eQTL. If a cis-eQTL and QTL were co-located in the same interval, the genes within the interval were identified as candidate functional QTL genes.

### Correlations between expression levels of candidate genes and shrimp body weights

The correlations between expression levels of candidate QTL genes and shrimp body weights were analyzed using linear regression and Pearson’s correlation analysis using the SPSS software program (version 24; https://www.ibm.com/cn-zh/spss).

### RNA interference of candidate genes

To verify the roles of candidate genes in growth rates, silencing of candidate genes was conducted by selecting a ∼500 bp sequence region within the conserved region of the candidate gene sequence to serve as a template for dsRNA synthesis. Specific primers (Supplementary Material Table S1) were designed using the cDNA of *L. vannamei* as the template, and the T7 RiboMAX Express RNAi System (Promega) was used to synthesize dsRNA in vitro. dsRNA of the enhanced green fluorescent protein gene (dsRNA-egfp) (Supplementary Material Table S1) was also synthesized for use in the experimental control group. The synthesized dsRNA was diluted and evaluated with agarose gel electrophoresis.

Shrimp used for silencing experiment were from a family that originally derived from an SPF strain obtained from the Hawaii Institute of Oceanography. The shrimp individuals were divided into four treatment groups and two control groups, with 50 shrimp in each group. Shrimp in the four treatment groups were injected with dsRNA for the four candidate genes at a dosage of 20 µg dsRNA per shrimp (diluted with 0.9% NaCl). The two control groups were injected with either dsRNA-egfp (diluted with 0.9% NaCl) or 0.9% NaCl at a dosage of 20 µg per shrimp. Muscle tissue was then collected at 0, 24, 48, and 72 h after injection and RNA was extracted to evaluate the expression of relevant genes at the mRNA level via real-time PCR. The real-time PCRs were conducted using the RR820A kit (TaKaRa) following the manufacturer’s instructions. The 18 S RNA gene was used as the internal reference, based on amplification with the primers 18s-F and 18s-R (Supplementary Material Table S1). The mRNA transcript levels were detected using a 7500 Fast fluorescence quantitative PCR instrument (ABI), with a reaction program comprising 40 cycles of 95 °C preheating for 30 s, 95 °C for 3 s, and 60 °C for 30 s. Three replicates were used for each tissue and the relative mRNA expression levels were calculated using the 2-∆∆CT method [28]. Statistical significance was evaluated via *t*-tests. After the initial injection, the initial weight of each shrimp was measured and the shrimp were fed for another month. dsRNA was injected every 10 days during this period. Each group of shrimp was fed four times a day at equivalent levels. Additionally, a shrimp strain (LV-B) with relatively high growth rates and a shrimp strain (LV-S) with relatively low growth rates were selected to evaluate gene expression profiles. Thirty shrimps were randomly collected from each strain. The weights of each group of shrimp were measured after one month and significant differences among groups were compared via *t*-tests using the SPSS software program (version 24; https://www.ibm.com/cn-zh/spss).

## Results

### Construction of genetic linkage map based on transcriptomes

A total of 270 cDNA libraries were constructed from the muscle tissues of two *L. vannamei* parent and 268 offspring individuals that were sequenced on the Illumina platform. After removing adapter sequences, low-quality sequences, and short sequences from the muscle tissue libraries, a total of 1,356.54 Gbp clean data were obtained comprising 8.56 Gbp on average from each parent and 4.01 Gbp on average from each offspring. The clean data from each sample was aligned to the *L. vannamei* genome, with mapping rates ranging from 47.24 to 72.91%.

**Fig. 1:**
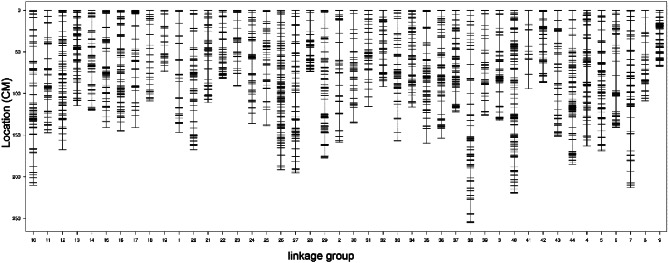
The high-density linkage map of L. vannamei based on RNA sequencing, illustrating the genetic distances between SNP markers. SNP markers are represented by black lines

To construct a genetic linkage map, single nucleotide polymorphisms (SNPs) were first identified from the read-mapped datasets. A total of 155,461 SNP tags were identified, including 134,808 polymorphic SNP tags, 27,486 genotypable tags, and 24,252 tags that could be used for genetic map construction. A high-density genetic linkage map was constructed from the SNPs containing 5533 markers spanning 44 linkage groups (Fig. 1). The total distance of the map was 6205.75 cM, the average marker interval was 1.12 cM, and the estimated completeness of the markers on the map was 99.89% (Supplementary Material Table S2). To validate the map, a sequence similarity Blastn search was conducted between 4053 mapping sequences from the map and our previously published genome of *L. vannamei* [24]. In total, 3890 hits were returned. According to the Blastn results, 44 linkage groups in the map were aligned to 44 pseudochromosomes of the genome, and the number of linkage groups was consistent with that of the previously reported maps of *L. vannamei* [29–31], confirming the successful construction of the map.

### QTL analysis

**Fig. 2:**
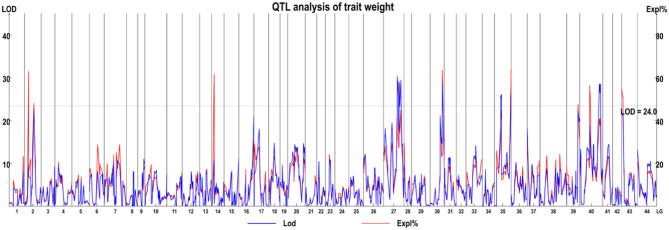
QTL analysis for body weight in *L. vannamei*. The blue and red traces represent the logarithm of odds (LOD) values and the contribution rate of the linkage, respectively. The gray line indicates the LOD threshold (24.0; *p* = 0.05). The numbers on the x-axis indicate the linkage group identifier

To identify QTLs associated with *L. vannamei* growth rate, the body weights of 268 individual shrimp were used as an indicator of growth rate. The average weight of the shrimp was 22.36 ± 3.72 g (Supplementary Material Table S3). To obtain significant QTLs, strict logarithm of the odds (LOD) thresholds was used (24.0), with a total of 11 QTLs being significantly correlated with weight traits and distributed on nine linkage groups (Fig. 2). Within these QTL intervals, 50 genes were included, containing 75 SNP markers (Supplementary Material Table S4).

### eQTL analysis

**Fig. 3:**
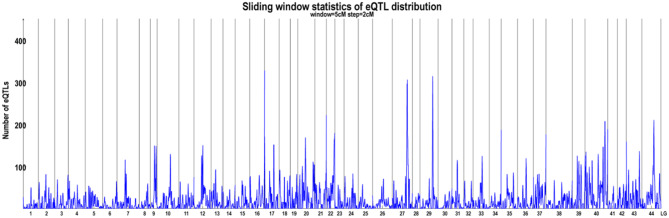
The distribution of eQTLs. The blue trace represents the number of identified eQTLs and the numbers on the x-axis indicate linkage group identifiers

To perform eQTL mapping, each gene was aligned to the *L. vannamei* genome to quantify expression, comprising a total of 19,641 genes. After removing genes with missing expression values, the remaining 11,503 genes were subjected to eQTL analysis. A total of 117,525 eQTLs were identified, including 12,088 trans-eQTLs and 105,437 cis-eQTLs that were widely distributed across different linkage groups (Fig. 3).

### Co-localization of QTLs and eQTLs

**Fig. 4:**
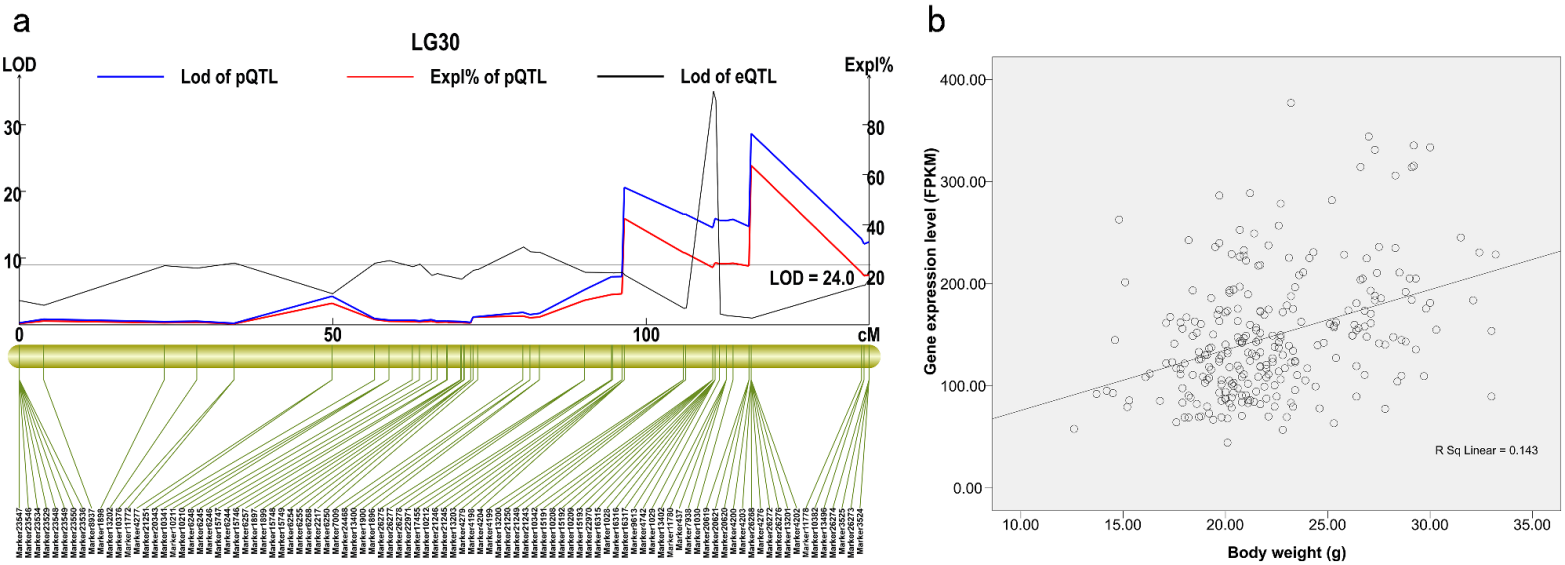
Co-location and regression analyses for the candidate trait gene c10972/f3p0/1885. (**a**) The cis-eQTL of c10972/f3p0/1885 gene overlapping with QTL intervals on linkage group 30. The blue trace represents the LOD values of the QTL for the linkage, while the red trace represents the contribution rate of the QTL for the linkage. The gray trace represents the LOD values of eQTLs of the linkage. (**b**) Regression analysis for the correlation between expression levels of the c10972/f3p0/1885 gene and the body weights of 268 *L. vannamei* used for genetic linkage map construction

Genes whose cis-eQTLs overlapped with the 11 weight-related QTL regions were first screened, leading to the identification of 7 such genes (Table 1; Fig. 4a). Correlations between the expression levels of these genes and shrimp weights were then conducted (Supplementary Material Table S5). The expression levels of four genes were significantly and positively correlated with the body weights of 268 shrimp that were used for genetic linkage map construction. In addition, two genes were negatively correlated with body weights, and one did not exhibit a significant correlation (Table 1; Fig. 4b). The four positively correlated genes (2329_0|path2, 5952_686|path0, C10972/f3p0/1885, and 545_0|path20) were consequently considered candidate genes related to the growth rates of shrimp. Annotation by comparison to the NR database indicated that the genes encoded “40S ribosomal protein S5”, “protein SMG7”, “metalloreductase STEAP4-like (Steap4)”, and “nascent polypeptide-associated complex alpha” products.

**Table 1:** Genes with cis-eQTLs overlapping with QTL intervals

Gene ID	NR annotation	QTL ID	Linkage group	Location of the Linkage group	Pearson’s *R*	*p*-value
2329_0|path2	40 S ribosomal protein S5	QTL2	LG16	144.63	0.248**	0
5952_686|path0	Protein SMG7	QTL4	LG27	151.19	0.212**	0
c10972/f3p0/1885	Metalloreductase STEAP4-like	QTL7	LG30	116.74	0.379**	0
545_0|path20	Nascent polypeptide-associated complex alpha	QTL11	LG43	12.432	0.218**	0
c10043/f1p0/1004	Probable small nuclear ribonucleoprotein	QTL6	LG30	96.502	−0.129*	0.035
c15091/f1p2/1469	Hypothetical protein X975_01914, partial	QTL9	LG39	121.69	−0.089	0.147
5952_5224|path0	Glutaryl-CoA dehydrogenase, mitochondrial	QTL10	LG40	194.27	−0.496**	0

*Note*: Pearson’s correlation values were used to evaluate the correlation between gene expression levels and shrimp body weight. **: correlation is statistically significant at the 0.01 level (two-tailed *t*-test)

### Silencing of candidate genes

**Fig. 5:**
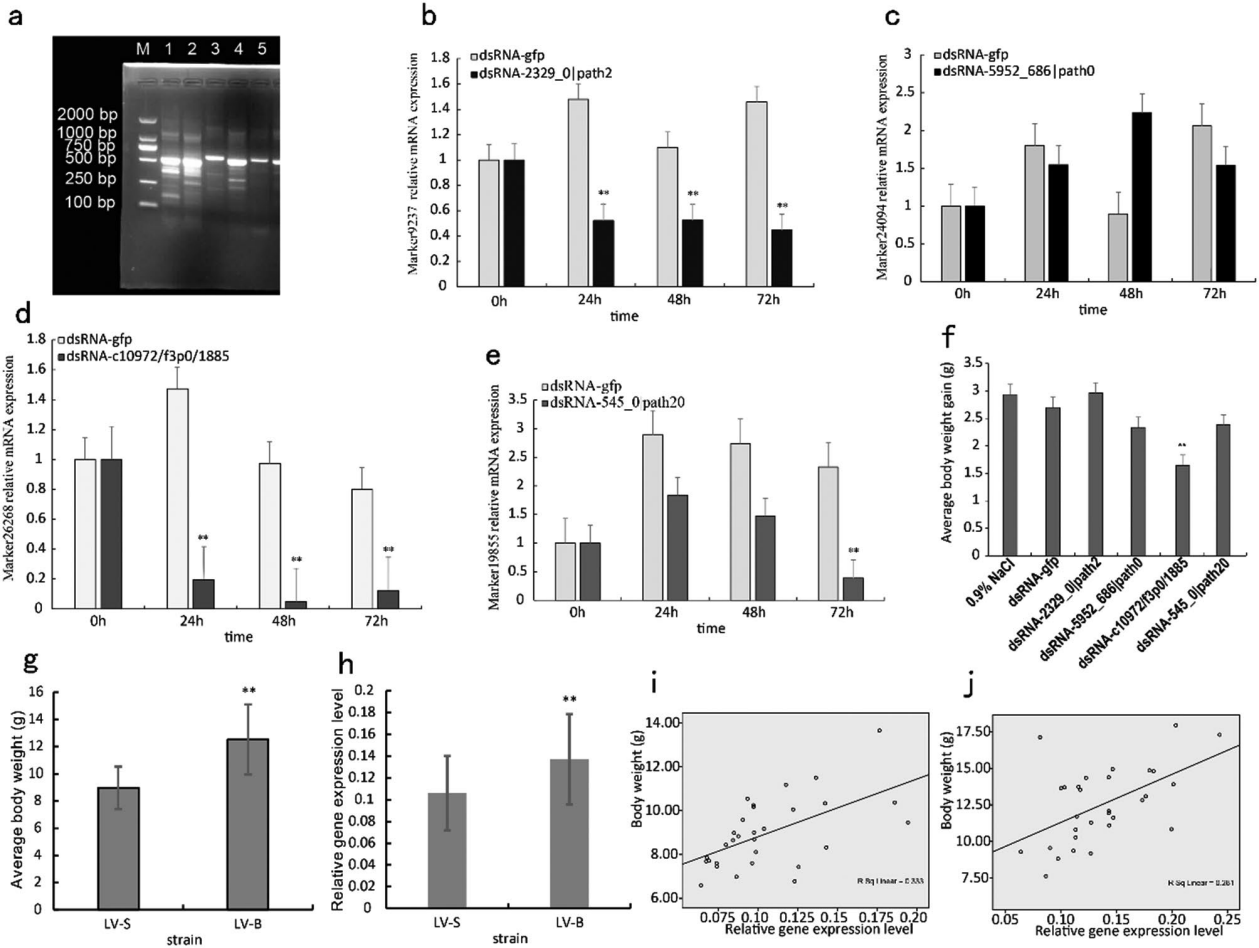
Silencing of candidate trait genes in *L. vannamei* by RNA interference. (**a**) Agarose gel electrophoretogram showing in vitro dsRNA synthesis. M: DNA Marker 2000; lane 1: dsRNA-2329_0|path2; lane 2: dsRNA-5952_686|path0; lane 3: dsRNA-C10972/f3p0/1885; lane 4: dsRNA-545_0|path20; lane 5: dsRNA-egfp. mRNA levels of different dsRNAs injected into shrimp, including (**b**) dsRNA-2329_0|path2, (**c**) dsRNA-5952_686|path0, (**d**) dsRNA-C10972/f3p0/1885, and (**e**) dsRNA-545_0|path20. mRNA levels are shown in comparison to the control group injected with dsRNA-egfp at 0, 24, 48, and 72 h after injection. (**f**) Comparison of weight gain in *L. vannamei* 30 days after injection with different dsRNAs. The data were statistically analyzed using paired Student’s *t*-tests. Asterisks denote statistical significance (*: *p* < 0.5; **: *p* < 0.01). (**g**) Comparison of average body weight between LV-B and LV-S shrimp strains. The data were statistically analyzed using paired Student’s *t*-tests. Asterisks denote statistical significance (**: *p* < 0.01). (**h**) Comparison of the average expression level of *Steap4* genes in LV-B and LV-S shrimp strains. The data were statistically analyzed using paired Student’s *t*-tests. Asterisks denote statistical significance (**: *p* < 0.01). (**i**) Correlation between individual body weight and the expression level of *Steap4* genes in LV-B strain. (**j**) Correlation between individual body weight and the expression level of *Steap4* genes in LV-S strain

To explore whether the aforementioned candidate genes were related to shrimp growth, the corresponding dsRNA for each gene were synthesized. The synthesized dsRNAs were evaluated using agarose gel electrophoresis after dilution, confirming their expected sizes (Fig. 5a). Gene expression was then interrupted by injecting the dsRNAs into shrimp, and samples were collected at 0, 24, 48, and 72 h after injection for real-time PCR analysis of mRNA. The expression of two candidate genes in the experimental group (2329_0|path2, Fig. 5b; and c10972/f3p0/1885, Fig. 5d) was significantly affected, while the expression of the other two genes was not significantly inhibited (Fig. 5c, e). Shrimp in the experimental, dsRNA-egfp, and control groups were raised for 30 days, and their body weights were evaluated accordingly (Supplementary Material Table S6). The average weight gain of shrimp injected with dsRNA-c10972/f3p0/1885 was significantly higher than that of the control groups injected with dsRNA-egfp or 0.9% NaCl (Fig. 5f). However, no significant differences in weight gain were observed among shrimp in groups injected with dsRNA corresponding to the other three candidate genes (Fig. 5f). Thus, shrimp growth was inhibited after silencing the c10972/f3p0/1885 gene (*Steap4*), suggesting that this gene is closely related to shrimp growth.

To further investigate the correlation between *Steap4* gene expression levels and shrimp growth rate, two shrimp strains with different growth rates (Fig. 5g) were selected to evaluate gene expression profiles. Thirty shrimps were randomly collected from each strain, RNAs were extracted, and the expression levels of the *Steap4* gene were measured using fluorescent quantitative PCR (Supplementary Material Table S7). Significant differences in the average expression levels of *Steap4* gene were observed between the two different *L. vannamei* strains. The average expression levels of the *Steap4* gene in the strain (LV-B) with high growth rates were statistically significantly higher than in the strain (LV-S) with low growth rates (Fig. 5h). Moreover, a significant correlation was observed between individual body weight and the expression level of *Steap4* gene in both LV-B and LV-S strains (Pearson’s *R* = 0.530, *p* = 0.003, and Pearson’s *R* = 0.577, *p* = 0.001, respectively) (Fig. 5i, j). Therefore, the expression of the *Steap4* gene is an important factor influencing the growth rate of *L. vannamei*.

## Discussion

The development of high-throughput sequencing technologies has led to an increase in the number of genetic linkage maps constructed using genotyping-by-sequencing methods [32]. This method involves sequencing genomic DNA, identifying SNPs, and constructing linkage maps [32]. In this study, we utilized RNA sequencing to construct a SNP linkage map for *L. vannamei* and simultaneously measure gene expression levels for eQTL analysis. Previous eQTL analyses primarily relied on gene chips [33], which are limited for non-model organisms. Therefore, we used RNA sequencing to measure gene expression levels instead. Transcriptomic sequencing was conducted on 270 individuals of the mapping population of *L. vannamei* to evaluate gene expression levels. This enabled the identification of abundant SNPs, which were used to construct a high-density genetic linkage map of *L. vannamei* with an average marker distance of 1.12 cM. Our map had a higher marker density compared to previous genetic maps of *L. vannamei* constructed using the amplified fragment length polymorphism (AFLP) or simple sequence repeats (SSR) markers, where the average marker distance was 7.6–15.1 cM [34, 35]. The density of our map was slightly lower than previous maps of *L. vannamei* constructed using specific length amplified fragment sequencing (SLAF-seq) method, where the average marker distance was 0.37–0.4 cM [36, 37]. However, a specific advantage of our map is its ability to obtain gene expression levels for eQTL analysis.

Using the constructed genetic linkage map, we identified 11 QTLs associated with *L. vannamei* growth rates. However, these QTL regions contained hundreds of genes. To further identify genes potentially responsible for growth rates, we conducted genome-wide eQTL analysis. This analysis revealed seven cis-eQTLs of genes that overlapped with the 11 QTLs. Four of these genes showed significant and positive correlations with shrimp body weight, suggesting their potential involvement in growth rates. Subsequent RNA interference experiments confirmed that only the expression of the *STEAP4* gene significantly affected growth rates. Previous studies have suggested a relationship between *STEAP4* and the growth of the red swamp crayfish (*Procambarus clarkii*), where *STEAP4* expression was higher in larger populations of *P. clarkii* compared to smaller populations [38]. Additionally, larger *P. clarkii* populations had higher lipid contents, indicating that *STEAP4* promotes growth rates by regulating lipid metabolism [38]. *STEAP4* belongs to the six transmembrane epithelial antigen of prostate (STEAP) family and is involved in regulating iron and copper homeostasis within cells [39]. It has also been implicated in the regulation of glucose and lipid metabolism in various tissues, such as adipose tissue, liver, muscle, and pancreatic beta-cells, by facilitating insulin-mediated glucose uptake and storage [40]. Studies in mice and humans have suggested that alteration of *STEAP4* expression may contribute to metabolic disorders like obesity, insulin resistance, and type 2 diabetes [40]. In our study, a combination of QTL and eQTL analyses indicated that *STEAP4* is related to *L. vannamei* growth, which was confirmed by gene silencing and shrimp growth experiments. Given the role of *STEAP4* in fat metabolism, we propose that it promotes the growth rate of *L. vannamei* by regulating fat metabolism. Therefore, *STEAP4* could be considered a gene associated with shrimp growth rates.

## Conclusions

In conclusion, we generated a genetic linkage map of *L. vannamei* and conducted comprehensive QTL and eQTL analyses across the entire genome. These analyses revealed the presence of a gene closely associated with shrimp growth rates, providing valuable genetic resources for shrimp breeding.

### Electronic supplementary material

Below is the link to the electronic supplementary material.

Supplementary Material 1

Supplementary Material 2

Supplementary Material 3

Supplementary Material 4

Supplementary Material 5

Supplementary Material 6

Supplementary Material 7

## Data Availability

RNA-seq reads for the QTL and eQTL analyses was deposited in the NCBI GenBank database under the accession PRJNA545592.
